# Contextualizing aging clocks and properly describing biological age

**DOI:** 10.1111/acel.14377

**Published:** 2024-10-11

**Authors:** Adiv A. Johnson, Maxim N. Shokhirev

**Affiliations:** ^1^ Tally Health New York City New York USA

**Keywords:** aging biomarker, aging clock, biohorology, biological age, chronological age, mortality

## Abstract

Usage of the phrase “biological age” has picked up considerably since the advent of aging clocks and it has become commonplace to describe an aging clock's output as biological age. In contrast to this labeling, biological age is also often depicted as a more abstract concept that helps explain how individuals are aging internally, externally, and functionally. Given that the bulk of molecular aging is tissue‐specific and aging itself is a remarkably complex, multifarious process, it is unsurprising that most surveyed scientists agree that aging cannot be quantified via a single metric. We share this sentiment and argue that, just like it would not be reasonable to assume that an individual with an ideal grip strength, VO_2_ max, or any other aging biomarker is biologically young, we should be careful not to conflate an aging clock with whole‐body biological aging. To address this, we recommend that researchers describe the output of an aging clock based on the type of input data used or the name of the clock itself. Epigenetic aging clocks produce epigenetic age, transcriptomic aging clocks produce transcriptomic age, and so forth. If a clock has a unique name, such as our recently developed epigenetic aging clock CheekAge, the name of the clock can double as the output. As a compromise solution, aging biomarkers can be described as indicators of biological age. We feel that these recommendations will help scientists and the public differentiate between aging biomarkers and the much more elusive concept of biological age.

## Graphical abstract

Biological age is an abstract concept that helps explain how an individual is functionally aging. Given that aging is a remarkably complex and multifarious process, it is infeasible to quantify biological age with a single aging biomarker. Rather than conflating a given metric with biological age, it makes more sense to conceptualize aging biomarkers, such as grip strength, VO_2_ max, or next‐generation aging clocks, as providing unique insights into different aspects of aging.

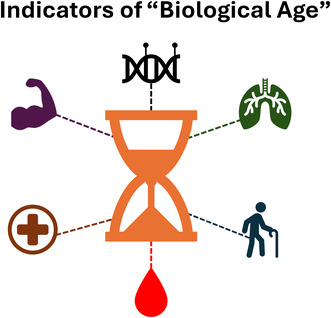

Usage of the phrase “biological age” has become increasingly common among biomedical scientists, as evinced by the increased prevalence of this term over time (Figure 1). The earliest record indexed in PubMed dates back to 1949 and, although a slight increase was observed in the late 20th century, this terminology really picks up in the second decade of the 21st century (Figure 1). This is not particularly surprising given that the first epigenetic aging clocks, models that estimate chronological age given methylomic data, were published by Bocklandt et al. in 2011 (Bocklandt et al., 2011) as well by Hannum et al. in early 2013 (Hannum et al., 2013) and by Horvath in late 2013 (Horvath, 2013). A manual examination of the literature reveals that this increased usage is largely due to the more frequent appearance and discussion of aging clocks. Several recent studies, for example, use the phrase “biological age” to describe the output of aging clocks based on clinical markers (Etain et al., 2024), proteins (Argentieri et al., 2024), DNA methylation (Tomusiak et al., 2024), microRNA (Roig‐Genoves et al., 2024), metabolites (van Holstein et al., 2024), or microbiota (Wang et al., 2024). For a more detailed discussion of the aging clock field, otherwise known as biohorology, we recommend an excellent review by Galkin et al. (2020).

**FIGURE 1:**
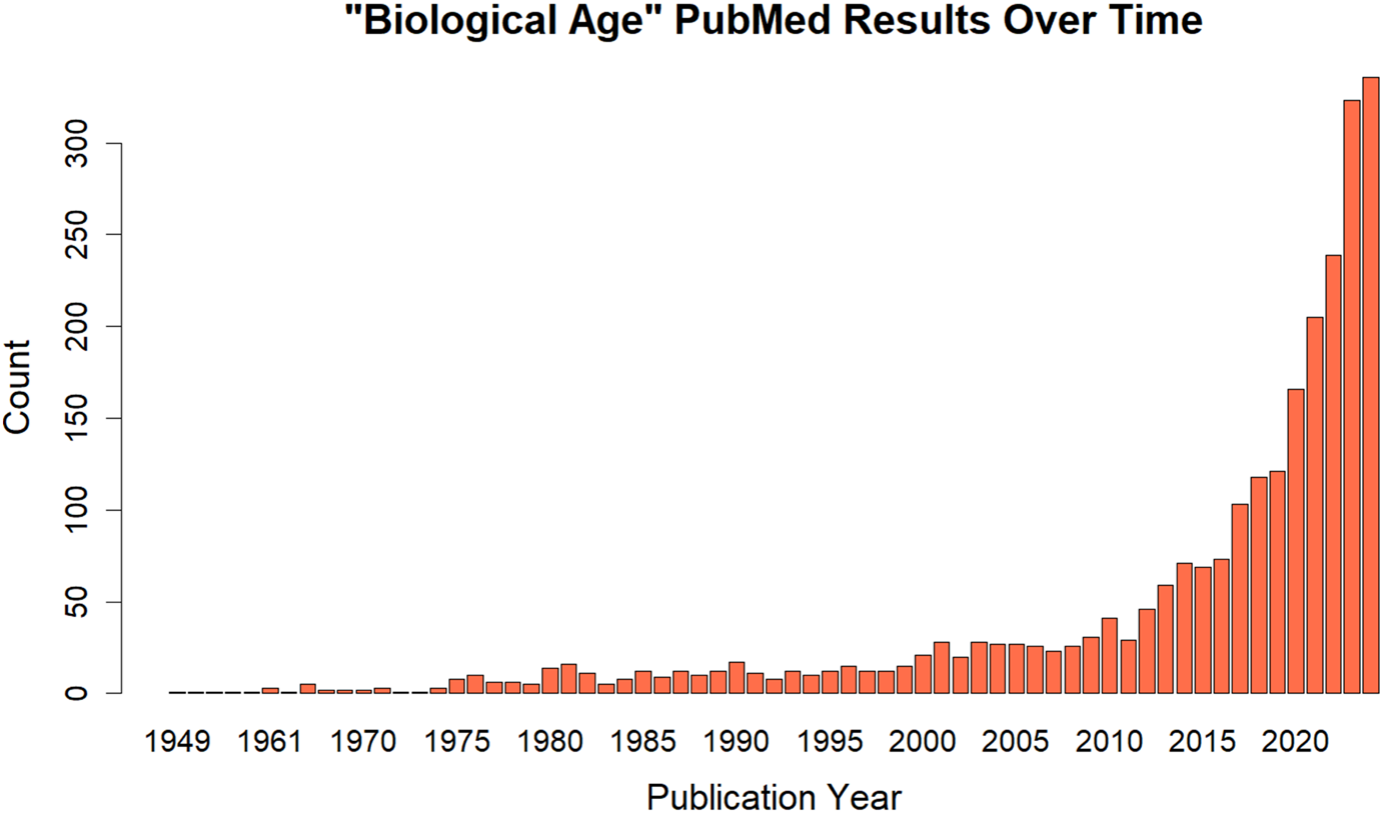
Usage of the phrase “biological age” in PubMed over time. On September 12th, 2024, all PubMed results returned from the search “biological age” were downloaded as a CSV file. Using R version 4.3.3 and RStudio version 2024.04.2, the CSV file was converted to a data frame and visualized using the built‐in barplot() function. This figure was specifically made by plotting publication count on the y‐axis and publication year on the x‐axis.

While it has become more prevalent for scientists to describe the output of an aging clock as biological age, this terminology may not be wholly appropriate. On the one hand, aging clock models are using biological signatures, specifically molecular expression data that tend to change with age in a predictable way in a population, to estimate chronological age. On the other hand, biological age has been described as something that is currently unmeasurable (Erema et al., 2022; Ferrucci et al., 2020) and as a more abstract, philosophical concept that explains how an individual is aging internally, externally, and functionally (Johnson et al., 2022). In this framework, biological age is an elusive mental model that helps us understand how functionally healthy an individual is relative to their chronological age. In line with this latter definition, surveyed longevity scientists generally do not feel that biological age can be measured via a single biomarker (Cohen et al., 2020). According to a survey done of scientists that participated in the *Biology of Aging Symposium: Understanding Aging to Better Intervene* in 2019, the majority of respondents (86%) indicated agreement with the statement “Aging cannot and should not be measured by a single metric because it is multi‐dimensional and heterogeneous.”

At this point, it is abundantly clear that the majority of molecular aging is tissue‐specific (Schaum et al., 2020; Shokhirev & Johnson, 2021; Slieker et al., 2018) and that the aging process is highly complex and multifarious (Lopez‐Otin et al., 2023). As such, it is not realistic to expect a single biomarker to be able to capture the full complexity of biological aging. The validated aging biomarker grip strength, for example, is reliably predictive of age‐related health outcomes, including mortality, in older adults (Leong et al., 2015). If we measure the grip strength of a 70‐year‐old adult directly using a dynamometer or indirectly by seeing how long the individual can perform a dead hang exercise, have we measured that person's biological age? If that individual's grip strength is above‐average relative to their chronological age, is it acceptable to say that the person is biologically young? The answer is, quite obviously, no. While a strong argument can be made for optimizing grip strength for longevity, it is very easy to imagine a scenario where someone has an excellent grip strength but is biologically old. As an example of this, someone might have remarkable grip strength but be an active smoker, drink heavily, and have suboptimal values of clinical aging biomarkers, such as fasting blood glucose, hemoglobin A1C, low‐density lipoprotein cholesterol, and high‐density lipoprotein cholesterol (Li et al., 2021).

The same is true for aging clocks, even the ones that qualify as validated aging biomarkers because they outcompete chronological age for predicting age‐related health outcomes. There are, for example, multiple next‐generation epigenetic aging clocks that track with mortality risk (Levine, 2020). If a next‐generation clock predicts that someone is epigenetically younger than their chronological age, is it reasonable to say that this person is biologically youthful and on track for a long, healthy life? While there is no doubt that the metric is predictive in longitudinal populations and is capturing an intriguing signal, we once again have to be cautious and acknowledge that no single metric can fully capture the complexity of biological aging. Moreover, it is possible to generate a large number of distinct aging clocks from the same set of molecular expression data. Given that myriad clocks can be constructed that produce discordant estimates (Porter et al., 2021), it is again unreasonable to conflate a single aging clock with whole‐body aging.

To address this, we propose a very simple solution. Biological age, on its own, should be exclusively described as an abstract concept and seen as something that is more qualitative. Aging clocks should be described based on the expression data they use as inputs or based on the name of the clock being utilized. In this paradigm, we would say that epigenetic aging clocks produce epigenetic age. Likewise, transcriptomic, proteomic, or metabolomic clocks respectively predict transcriptomic age, proteomic age, or metabolomic age. If a clock has a unique name, that name can double as the clock's output. Our recently developed epigenetic aging clock CheekAge (Shokhirev et al., 2024), for example, could be described as predicting either epigenetic age or CheekAge. Lastly, a compromise solution would be to describe an aging biomarker as an indicator of biological age. Here, each aging biomarker, whether it is grip strength (Leong et al., 2015), cardiorespiratory fitness (Mandsager et al., 2018), the frailty index (Kojima et al., 2018), an aging clock (Levine, 2020), or something else, is providing a unique insight into biological aging. By emphasizing that an aging biomarker is just an indicator, however, we convey that much of the aging process is still not being captured.

While these recommendations may seem nitpicky, we feel that adhering to these suggestions will allow scientists and the public to differentiate between a validated aging biomarker that has scientific utility and the much more complicated, still poorly understood process of whole‐body biological aging.

## AUTHOR CONTRIBUTIONS

AAJ and MNS both contributed to the design of this paper. AAJ wrote the paper and created the visuals while MNS performed editing.

## CONFLICT OF INTEREST STATEMENT

Both authors are full‐time employees of the company Tally Health (New York, New York, United States of America). The authors have no other conflicts of interest to declare.

## Data Availability

Data sharing is not applicable to this article as no new data were created or analyzed in this study.
